# Delayed Paraplegia by Thoracolumbar Fracture in Diffuse Idiopathic Skeletal Hyperostosis Developed After Femur Fracture Surgery: A Case Report

**DOI:** 10.7759/cureus.86036

**Published:** 2025-06-15

**Authors:** Masaaki Shiomi, Nobuaki Tadokoro, Yoshinori Satake, Katsuhito Kiyasu, Masahiko Ikeuchi

**Affiliations:** 1 Orthopedic Surgery, Kochi University, Kochi, JPN

**Keywords:** diffuse idiopathic skeletal hyperostosis, femur fracture, geriatric people, ground-level fall, osteoporosis

## Abstract

Diffuse idiopathic skeletal hyperostosis (DISH) increases with aging, as does osteoporotic fracture. DISH spine fractures are sometimes difficult to detect at the initial evaluation and result in neurological compromise. We report a case of unstable DISH spine fracture with neurological deficits developing from an asymptomatic DISH spine fracture. A 98-year-old independent female suffered from a right femoral shaft fracture due to a ground-level fall. After she started early mobilization following fracture surgery, she developed mild back pain and became paraplegic. Imaging studies showed DISH spine fracture at Th10 level with cord compression. She underwent posterior spinal fusion for the DISH spine fracture, but the muscle weakness in her lower limbs persisted. Unlike the painful and non-ambulatory femoral shaft fracture, DISH spine fractures present diagnostic challenges. Asymptomatic cases could result in delayed diagnosis with neurological deficits and other morbidities. Although CT with multiplanar reformatted imaging and MRI are useful for detecting subtle fractures, the requirements for spinal CT and MRI examination in low-energy trauma settings in patients without back pain remain controversial. The proactive diagnostic approach, consisting of physical evaluations for pain and neurological deficits and the complementary imaging studies, should be encouraged for the early detection of hidden spine injury in patients having DISH spine.

## Introduction

The number of geriatric people is dramatically increasing globally [1]. Many geriatric patients have osteoporosis and reduced physical function, which leads to fractures due to ground-level falls. Fractures associated with ground-level falls are very common in daily clinical practice. Diffuse idiopathic skeletal hyperostosis (DISH) is an idiopathic disease characterized by calcification and ossification of spinal ligaments and associated with aging [2,3]. Fractures within a DISH spine are notoriously unstable because of the stiff, ankylosed spine with long lever arms. Subtle fractures can advance severe three-column injury (an unstable spinal fracture that involves all anteroposterior spinal components) with neurological deficit. Physical symptoms such as back pain and neurological symptoms usually serve as the trigger to diagnose spine fractures. However, patients with DISH spine fractures posed a diagnostic challenge in case of preserved trunk support without symptoms such as pain and neurological deficits [4,5]. Thus, the delayed diagnosis is still problematic. However, detailed case reports of the delayed diagnosis are still scarce. On the other hand, femoral shaft fracture is hardly ever missed because of the inability to walk due to pain and loss of load-bearing capacity.

In this paper, we report a case of DISH spine fracture concomitant with femoral shaft fracture, focusing on the cause of the delayed diagnosis of DISH spine fracture.

## Case presentation

**Figure 1:**
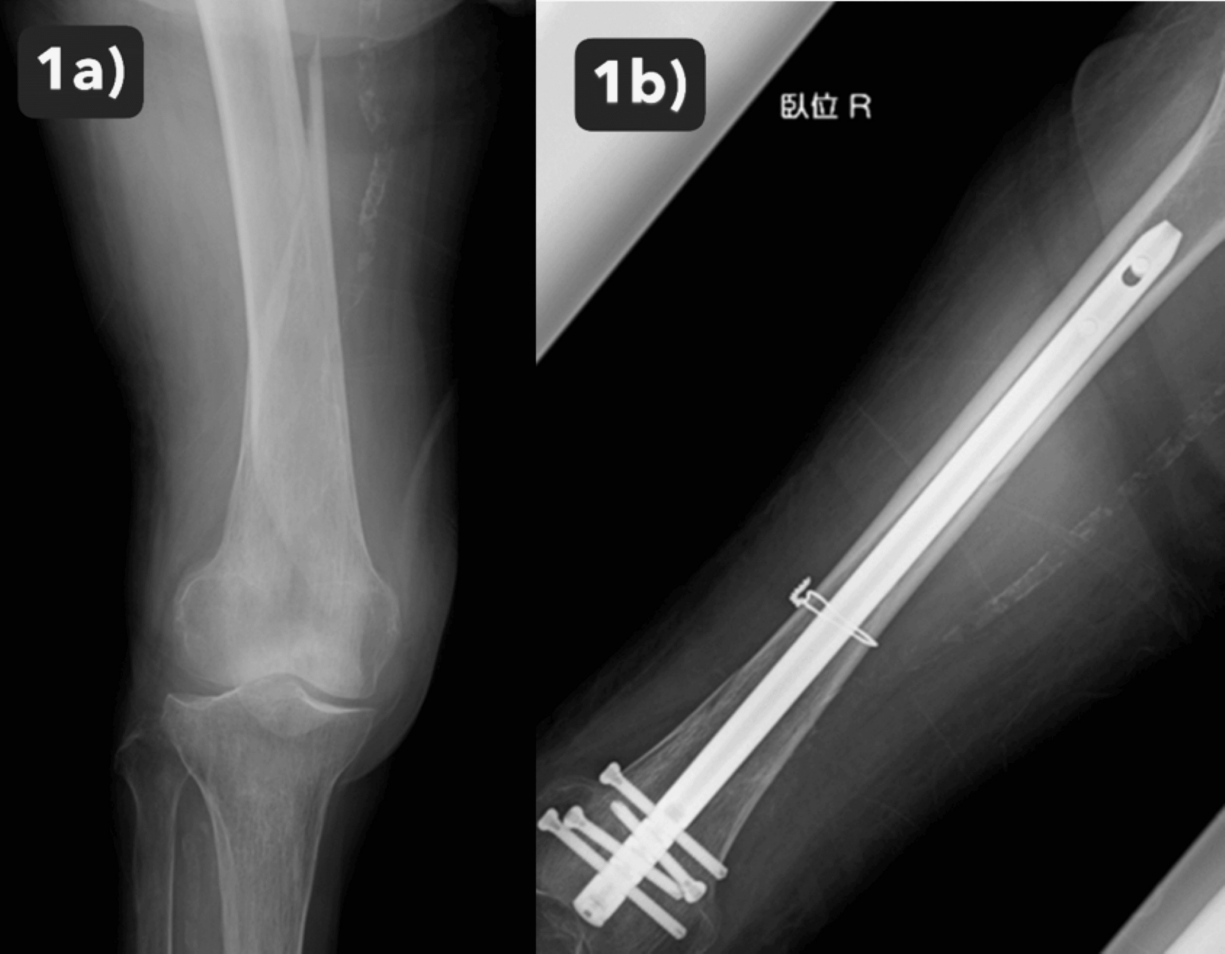
Right femur fracture at initial evaluation and postoperative radiograph of the right femur 1a) Right femur fracture at initial evaluation; anteroposterior and lateral radiographs showed the displaced right femoral shaft fracture. 1b) Postoperative radiograph of right femur; The fracture was fixed with a retrograde intramedullary nail with cerclage wiring.

The patient is a 98-year-old female. She fell from ground level at home and was rushed to our hospital with pain in her right thigh. Initially, she did not complain of pain in any other part of her body or any neurological deficit. Based on the results of the physical examination, X-ray, and CT scan, she was diagnosed with a right femoral shaft fracture (AO classification: 31A1) (Figure 1a). There were no significant medical comorbidities except for osteoporosis (YAM 52%) [6]. Despite the advanced age, the patient resided independently before injury. At admission, a CT scan from chest to pelvis was obtained to rule out pneumonia because of the COVID-19 pandemic. At this point, only horizontal CT images were reconstructed, and sagittal and coronal CT images were not reconstructed.

**Figure 2:**
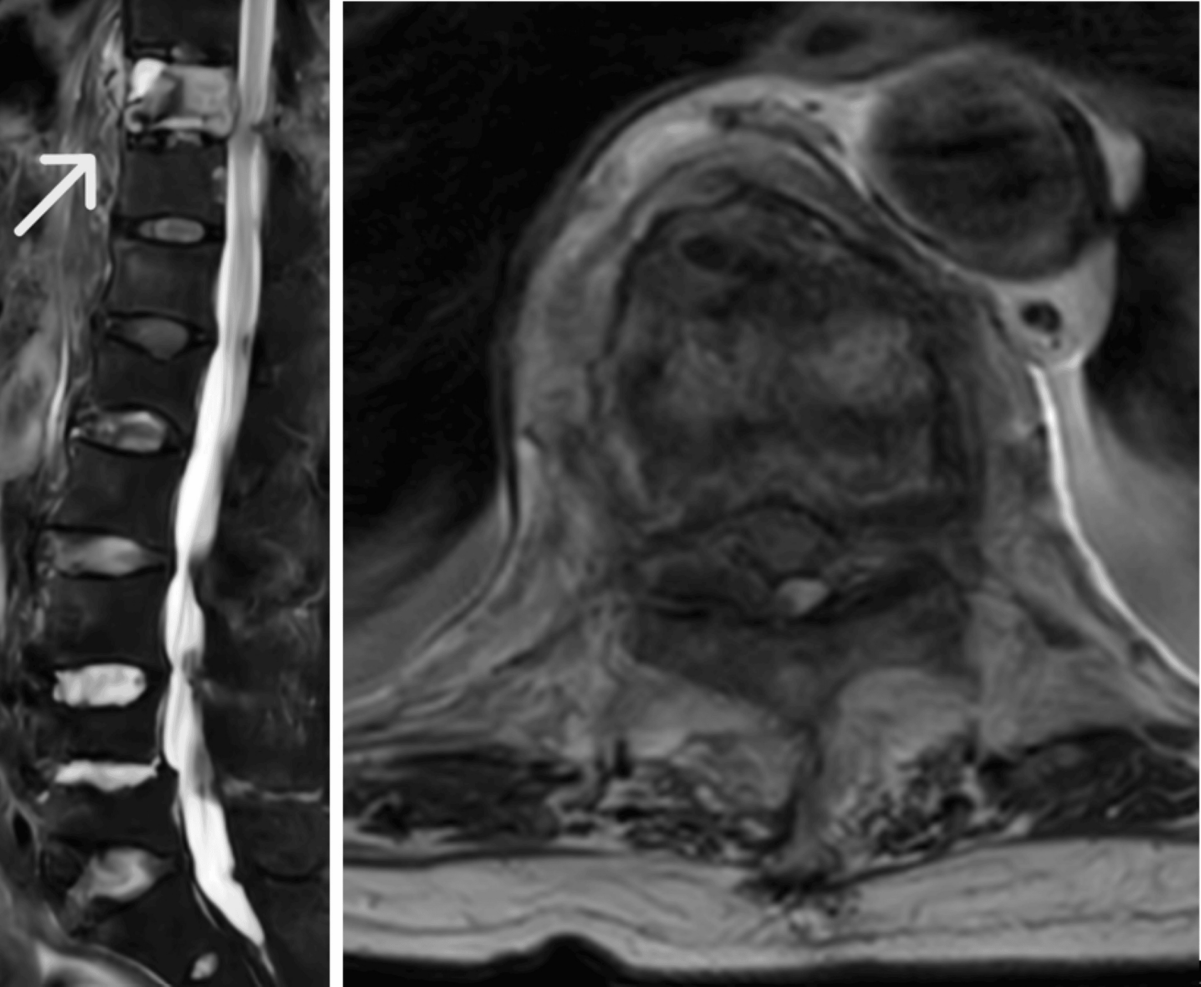
Magnetic resonance imaging (MRI) at diffuse idiopathic skeletal hyperostosis (DISH) spine diagnosis The sagittal STIR MRI showed the signal changes of the Th10 vertebral body and posterior ligaments suggesting acute fracture. The T2 axial view showed cord compression at T10/11 level and hematoma formation around the T10 vertebral body.

**Figure 3:**
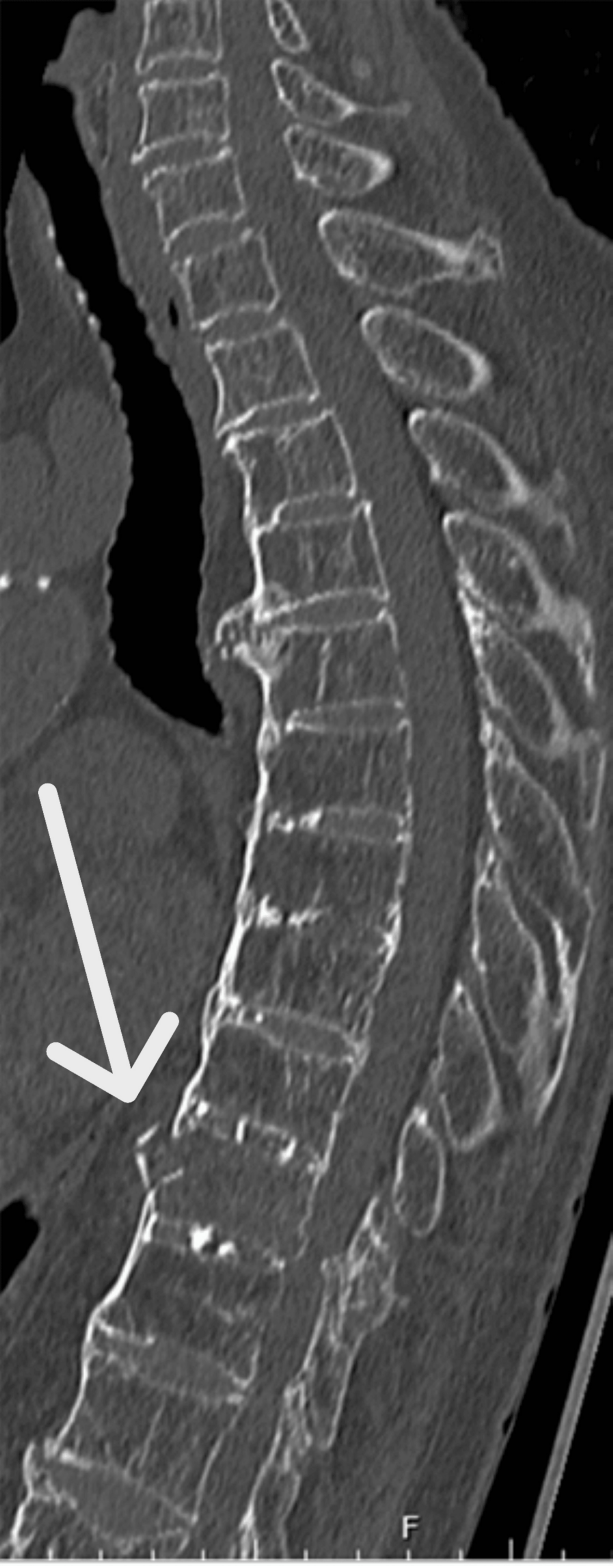
CT scans at diffuse idiopathic skeletal hyperostosis (DISH) spine diagnosis The sagittal CT depicted T10 anterior vertebral wall comminution and T10/11 canal stenosis due to the ossified yellow ligament.

**Figure 4:**
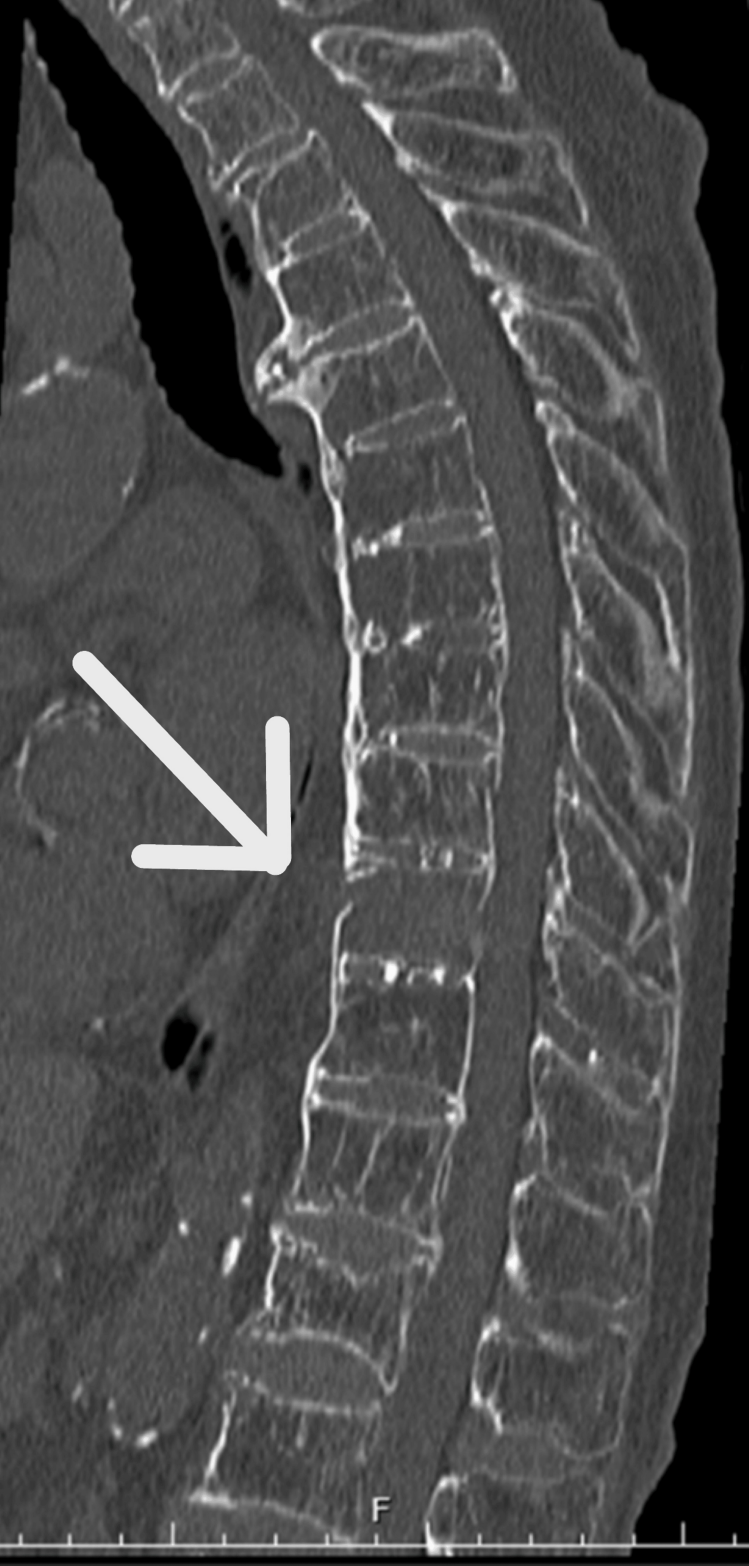
Sagittal reconstructed CT scan from the chest CT scan at the initial evaluation Discontinuity of the Th10 anterior vertebral wall was observed.

**Figure 5:**
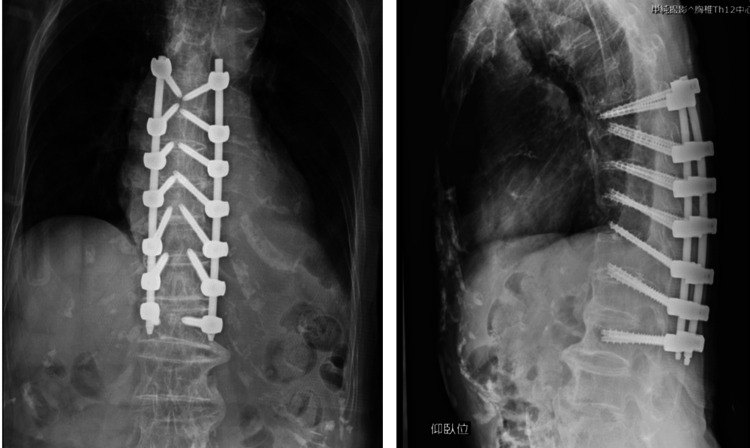
Postoperative spine Th10 fracture was fixed from T7 to L1 using double endplate penetrating screws.

The patient underwent open reduction and internal fixation with an intramedullary nail for the right femur fracture (Figure 1b). Postoperatively, the patient’s general condition was stable. Rehabilitation therapy was initiated on the day after surgery to optimize her functional recovery and mobility. She had no pain except for the injured right leg, soon after surgery. However, during the first postoperative week, the patient developed bilateral lower limb muscle weakness and became paraplegic. She also complained of mildly intense back pain. MRI imaging revealed a fracture of the Th10 vertebral body within the DISH region, with associated intramedullary signal changes suggesting spinal cord compression as the likely cause of paraplegia (AIS Grade A)(Figure 2). In addition, a CT scan showed comminuted anterior cortical bone and spinal canal stenosis due to an ossified yellow ligament (Figure 3). At this point, we reviewed the reconstructed sagittal images from the chest CT scan taken at admission and realized the slight discontinuity of the anterior Th10 vertebral body cortex (Figure 4). This fracture within DISH, which was initially minimally displaced without neurological deficits, was considered to have progressed to a highly unstable three-column injury and caused paraplegia from the CT findings of the initial and second CT. Despite conservative management, including bed rest, weakness persisted. Subsequently, the patient underwent thoracolumbar posterior fixation surgery (Figure 5), but remains paraplegic (AIS Grade A).

## Discussion

The prevalence of DISH in thoracoabdominal CT scans performed by departments other than orthopedic surgery is 27.2%, which is higher in elderly patients and is not uncommon [7]. Clinical information such as no back pain, minor trauma, no neurological deficit, and the presence of a femoral fracture unfortunately led to the delayed diagnosis of DISH spine fracture. The DISH spine fracture could result in severe neurological deficits and significant comorbidity. This case highlighted the importance of recognizing concomitant subtle DISH fractures even in a striking femoral shaft fracture with a ground-level fall.

Detecting DISH spine fractures in low-energy trauma settings is challenging. Patients having DISH spine have osteoporosis with a high prevalence [8]. Concomitant other extremity fractures complicated 39% of thoracolumbar fractures due to ground-level fall [9]. The perspective of possible multiple fractures in low-energy osteoporotic patient trauma is important. The ground-level fall and no neurological deficit at initial presentation were also reported as the risk factors for delayed diagnosis of DISH spine fracture4. Thus, identifying DISH spine seems important for detecting DISH spine fractures in low-energy trauma. Although spine CT and MRI are powerful tools to detect subtle fractures, it is difficult to apply CT and MRI to all trauma patients, considering the radiation exposures and medical costs. A chest radiograph taken upon admission may help to find DISH spine by vertebral body bony bridging. In addition, radiographic vertebral fractures and recent artificial intelligence advances are helpful in finding osteoporosis [10,11]. DISH spine and/or osteoporosis are feasible factors, considering additional advanced spine imaging studies such as CT or MRI. The whole-spine CT scan may be a more feasible imaging study in emergency conditions without neurological deficit than MRI [12-14]. However, it is crucial to use CT with multiplanar reformatted imaging (CT-MPR), including all axial, sagittal, and coronal views, to detect the non- or minimally-displaced fractures like in our case [4,5].

The pain symptoms in DISH patients are reported both to be strong [15] or weak [16]. Considering that more than two-thirds of osteoporotic vertebral compression fractures are asymptomatic [17], it may not be surprising that this 98-year-old patient initially had no back pain under right thigh pain. Although low back pain is a key to considering spine injury in clinical settings, no clear, routinely useful red flags for locating vertebral fractures exist [18]. Some asymptomatic spine fractures could be missed at initial presentation [4,5]. In addition to the whole-spine CT after the recognition of DISH spine, daily, ongoing tertiary survey, especially soon after the mobilization from this patient history, might be an early detection measure for hidden injury in geriatric, DISH spine, osteoporosis-associated trauma patients, as in polytrauma patients [19].

DISH spine fracture is highly unstable and susceptible to neurological injury, both at the time of presentation and later on [5]. Thus, DISH spine fracture is considered a surgical indication for stabilization [20]. Because this case had both long fused segments and spinal canal stenosis with an ossified yellow ligament, the instability at the fracture site directly resulted in neurological deficit.

## Conclusions

Elderly people with osteoporosis can suffer multiple fractures even if they fall at ground level. Particular attention should be paid to the DISH spine even with the existence of another fracture, because an asymptomatic fracture at initial presentation exists. This case highlights the possibility of delayed neurological deficits in patients with DISH spine fractures. Additional imaging studies such as CT-MPR and MRI, and ongoing physical surveys help in early detecting DISH spine fractures.
